# Two decades of end-of-life conditions of older adults: older and more protracted?

**DOI:** 10.1016/j.ssmph.2025.101858

**Published:** 2025-08-30

**Authors:** Dorly J.H. Deeg, H. Roeline W. Pasman, Martijn Huisman, Bregje D. Onwuteaka-Philipsen

**Affiliations:** a Amsterdam University Medical Centers, Vrije Universiteit Amsterdam, Department of Epidemiology and Data Science, Amsterdam Public Health Research Institute/Ageing and Later Life Program, P.O. Box 7057, 1007 MB, Amsterdam, the Netherlands; b Amsterdam University Medical Centers, Vrije Universiteit Amsterdam, Department of Public and Occupational Health, Amsterdam Public Health Research Institute/Ageing and Later Life Program, P.O. Box 7057, 1007 MB, Amsterdam, the Netherlands

**Keywords:** Older adults, End of life, Health conditions, Within-person change, Time trend

## Abstract

**Background:**

The mortality decline during the past decades has postponed the age at death. Dying at older ages may involve alterations in health trajectories at the end of life. This study examined 10-year period differences in level and changes in health conditions from 3 months to 3 days before death. Distinction was made between cancer and non-cancer decedents, because their trajectories are known to differ.

**Methods:**

Data were provided by proxies of participants in the Dutch population-based Longitudinal Aging Study Amsterdam, who died between 2005–2009 (midyear 2008) and 2017–2019 (midyear 2018), with complete data for 162 and 124 decedents, respectively. Health conditions included functional limitations, symptom severity, and low consciousness.

**Results:**

Average age at death increased from 81.0 (2008) to 83.5 (2018). 48 % of decedents were men. Cancer constituted 33 % of causes of death. Controlling for sex, age at death, and education, non-cancer decedents experienced more often low consciousness at 3 months before death in 2018 than in 2008. At 3 months in 2018, cancer decedents experienced fewer functional limitations than in 2008 and fewer than non-cancer decedents. In both periods, cancer decedents experienced steeper functional declines than non-cancer decedents. Trajectories of symptom severity were similar in cancer and non-cancer decedents in both periods.

**Discussion:**

In non-cancer decedents, but not in cancer decedents, the dying process was more protracted in 2018 than in 2008. Level and increase in symptom severity did not improve over time for both cancer and non-cancer decedents, suggesting that improvement of palliative care is warranted for both groups.

## Highlights

• In two nationally representative samples of decedents sampled in 2005–2009 and 2017–2019, age at death shifted upwards by 2.5 years.
• Trajectories of change in health conditions in the last 3 months of life as retrospectively reported by proxies, were more protracted in 2017–2019 than in 2005–2009 in non-cancer decedents.
• By contrast, caner decedents experienced fewer functional limitations in 2017-2019 than in 2005-2009.
• In both periods, cancer decedents experienced fewer functional limitations, but steeper functional declines than non-cancer decedents.
• Level and increase in symptom severity did not improve over time for both cancer and non-cancer decedents.

## 1 Introduction

The continued decline in mortality during the past decades has shifted the age at death towards older ages (Thatcher et al., 2010). Important reasons for this shift are substantial advances in diagnosis and treatment of several major chronic conditions. This development enables people with a chronic condition to live longer (Crimmins et al., 2019; Deeg et al., 2013). With increasing age, however, the risk of poor health and frailty increases (Jenkins et al., 2022). If the age at onset of poor health conditions and frailty does not increase, this may mean that a longer period of life will be spent in poor health and with frailty. In turn, a longer duration of poor health conditions and frailty increases the likelihood of more severe health conditions and higher levels of frailty at the end of life. This would imply that at higher ages at death, the end-of-life conditions are likely to be more protracted.

Whether the period of life spent in poor health increases or not, is generally studied using the (un)healthy life expectancy indicator, which is based on age-specific prevalences of good and poor health and population survival rates from a certain age onwards (Saito et al., 2014). Trends over time of unhealthy life expectancy show mixed results, depending on the health indicator used, the time period, and the country. However, most recent studies show an expansion of the years in poor health (Spiers et al., 2021). This is also true for the Netherlands, the country in which the current study is situated (Deeg et al., 2018).

In addition to a development towards higher ages at death, time trends in causes of death show changes over time. In Western countries in particular, mortality of both cardiovascular diseases and cancer have declined, but the decline in cardiovascular diseases was steeper than that in cancer (Aldridge & Bradley, 2017; GBD 2018). In several Western countries, including the Netherlands, cancer has become the largest cause of death (Eurostat, 2020; Statistics Netherlands, 2020). As shown by several studies, health impairments and health care use at the end of life show a more precipitous change for cancer than for other causes of death (Ebeling et al., 2023; Lunney et al., 2003; Murray et al., 2024; Stolz et al., 2024; Teno et al., 2001). At the population level, the proportional increase in cancer as a cause of death may imply a shortening of the time in poor health prior to death.

Thus, two concurring trends seem to influence the duration and complexity of poor health at the end of life: the increase in age at death and the proportional increase in cancer as a cause of death. Against the background of these trends, this study aims to examine changes in health conditions in the final three months of life across two periods (late 2000s and late 2010s), distinguishing cancer and other causes of death. We hypothesise that end-of-life conditions for causes of death other than cancer have become more protracted. For cancer decedents, according to existing evidence, we expect a more precipitous change in end-of-life conditions in the final three months compared to non-cancer decedents. However, cancer has been shown to have increasingly the characteristics of a chronic disease, as cancer patients survive to increasingly older ages due to expansion of preventive screening and advances in treatment (Crimmins et al., 2019; Geijteman et al., 2024; Payne & Kobayashi, 2021). Moreover, the co-existence of cancer and other conditions increases (Ritchie et al., 2011). Therefore, we expect that end-of-life changes of cancer decedents and non-cancer decedents have become more similar from the 2000s to the 2010s.

We study several health conditions, in order to obtain a comprehensive view. This choice of outcomes corresponds to a needs-based approach for eligibility for palliative care, as recommended by Kawashima and Evans (2023). First, we include functional limitations, which have relevance to the daily life of patients and have often been used in end-of-life trajectory studies (Gill et al., 2010; Lunney et al., 2003; Smith et al., 2013; Stolz et al., 2021; Teno et al., 2001). Second, we include severity of four physical and mental symptoms, i.e., pain, shortness of breath, fatigue, and anxious or depressed mood, as these are particularly relevant to palliative care (Cheraghlou et al., 2016; Fleming et al., 2017; Gill et al., 2021; Kristensen et al., 2022; Singer et al., 2015; Versluis et al., 2024). Third, as low consciousness frequently occurs near the end of life and has implications regarding treatment options (Fleming et al., 2017; Kaspers et al., 2013), we include level of consciousness.

## 2 Methods

### 2.1 Study sample

The data were derived from deceased participants in the Longitudinal Aging Study Amsterdam (LASA), an ongoing, cohort-sequential study consisting of three successive cohorts with baseline measurement waves in 1992/93, 2002/03, and 2012/13 (Hoogendijk et al., 2025). These cohorts are based on samples of older adults, drawn from 11 municipal population registries in three regions in the Netherlands. These regions reflect the national distribution of urbanisation and population density and together represent the socio-cultural variety in the Netherlands. The first cohort's initial ages were 55–85 years; in this cohort, higher ages and men were oversampled. The baseline cooperation rate was 62 %, resulting in a sample size of 3107. The second and third cohort were aged 55–64 years, with sample sizes 1002 and 1023 participants, and similar cooperation rates of 62 % and 63 %, respectively. These cohorts were merged with the earlier cohorts at their first follow-up. Every three or four years, face-to-face interviews were conducted in the home of the participants by trained interviewers. Informed consent was obtained from every participant. During the interviews, the participants were asked to name two proxy respondents in case the participants themselves could not be contacted.

The representativeness of the LASA cohort was examined by comparing mortality rates in the LASA sample to that in the Dutch population (Hoogendijk et al., 2025). Although mortality rates were slightly higher in the general population compared to the LASA sample, for most age-sex groups the differences did not exceed 1 % point. This means that mortality rates in the LASA sample are not substantially different from mortality rates in the Dutch general older population.

For the current study, data were provided by proxies of participants who had died between 2005 and 2009 (‘sample 2008’, termed after the midyear) and between 2017 and 2019 (‘sample 2018’) and who had given permission to contact a named proxy. Excluded were participants who had refused participation in the waves preceding their death. If a proxy had not provided family care in the last three months of the decedent, contact information was requested from the person who had provided care (if applicable). In 2005–2009, 311 participants fulfilled the inclusion criteria, of which 284 proxies were approached, since 27 proxies (10 %) were not found; 167 proxies (59 % of 284) completed and returned the questionnaire. In 2017–2019, 215 participants fulfilled the inclusion criteria, of which 161 proxies could be contacted; 131 proxies (81 % of 161) completed and returned the questionnaire. For this study we excluded participants who died due to an accident in the absence of morbidity or frailty, resulting in 162 and 124 participants in samples 2008 and 2018, respectively.

In sample 2008, 82 % of the proxies were a child of the decedent and 9 % were the decedent's partner. In sample 2018, 69 % of the proxies were a child of the deceased and 16 % were the decedent's partner. The remaining proxies were siblings, other family, or friends (Table 1).

**Table 1:** Characteristics of decedent samples in two periods, ages at death 60 and over; weighted to the age-at-death and sex distribution of decedents in the general population in each mid-year (2008 and 2018).

	2005–2009	2017–2019	p-value
N	162	124	
Relationship of deceased to proxy (%):			0.003
Partner	8.6	15.8	
Parent	82.1	69.2	
Brother/Sister	1.2	7.5	
Friend/Non-kin	5.6	1.7	
Other	2.5	5.8	
Year of death^a^ (M, sd)	2006.9 (1.3)	2018.1 (0.8)	n.a.
Age-at-death^a^ (M, sd)	81.0 (9.5)	83.5 (8.9)	0.064
Gender (% male)	47.5	48.4	0.886
Education in years (M, sd)	9.1 (3.1)	10.2 (3.6)	<0.001
Partner (yes, %)	46.3	51.3	0.411
Place of death (%):			0.011
Own home	31.7	35.8	
Residential home	14.3	19.2	
Nursing home	20.5	18.3	
Hospital	28.0	13.3	
Hospice	2.5	10.0	
With family	–	0.8	
Outside	3.1	2.5	
Cause of death (%):			0.479
Cancer	30.1	36.1	
Dementia	8.0	10.9	
Cardiovascular disease	15.3	15.1	
COPD	1.2	3.4	
Diabetes	2.5	1.7	
Stroke	11.0	4.2	
Fall accident	2.5	4.2	
Pneumonia	1.8	0.8	
Unclear	8.0	8.4	
Old age	14.1	9.2	
Other	5.5	5.9	
Functional limitations at 3 months^b^ (M,sd)	12.0 (6.6)	11.2 (6.3)	0.363
Functional limitations at 3 days^b^ (M, sd)	16.6 (6.3)	17.0 (5.4)	0.641
Symptom severity at 3 months^c^ (M, sd)	3.3 (1.9)	3.3 (1.7)	0.816
Symptom severity at 3 days^c^ (M, sd)	4.6 (2.3)	4.3 (1.9)	0.540
Low consciousness at 3 months (%)	6.5	12.2	0.106
Low consciousness at 3days (%)	37.6	38.5	0.894

a From population registry.

b Range 0 (no limitations) to 20 (most limited).

c Range 0 (no symptoms) to 8 (most severe).

### 2.2 Measures

Data were obtained through a written questionnaire in 2010 and 2020 for samples 2008 and 2018, respectively. The questionnaires consisted of structured questions about socio-demographic characteristics of the decedents, cause of death, health conditions at 3 months and 3 days before death, and place of death. Age at death and sex were derived from the Netherlands population registry. Level of education in years and partner status were derived from the most recent LASA wave in which a decedent had participated.

Cause of death was derived from proxy-reports. In the written questionnaire, specific causes of death were listed, including cancer, dementia, cardiovascular diseases, chronic obstructive pulmonary diseases (COPD), diabetes, and stroke. The questionnaire also provided ample space for free text describing the cause and circumstances of the death, so that other causes than those listed could be reported. A separate question asked about any diseases that were present in the last phase of life. For this study, the underlying cause of death was assigned as much as possible according to the ICD-10 coding rules followed by Statistics Netherlands (World Health Organization 2011). The assignment was based on both proxy-reported causes and the free text, in addition making use of all diseases that were reported in the separate question, the age at death, and the place of death. The final list of causes of death included cancer, dementia, cardiovascular diseases, chronic obstructive pulmonary diseases (COPD), diabetes, stroke, fall accident, pneumonia, other specific cause, old age (mostly no clear disease, but failure in several organs), and unclear (mostly because of multimorbidity). In the analyses, a dichotomous variable was created to distinguish cancer (value 1) from non-cancer (value 0) deaths.

Health conditions included functional limitations, physical and mental symptoms, and low consciousness at 3 months and 3 days before death. Functional limitations were measured with questions about difficulty doing five activities, including going up and down a staircase, dressing and undressing, sitting down in and getting up from a chair, walking outside during 5 min without stopping, and using own or public transportation. Response options ranged from 0 to 4: ‘yes, without difficulty’ (0) to ‘not possible’ (4). The sum score ranged from ‘able to perform all activities of daily living without difficulty’ (0) to ‘unable to perform any activity’ (20).

Physical and mental symptoms at 3 months and 3 days before death included pain, shortness of breath, fatigue, and anxious or depressed mood. Severity of each symptom was rated on a 3-point scale: no symptom (0), moderately severe (1), severe (2). Symptom severity was calculated as the sum score and ranged from ‘no symptoms’ (0) to ‘maximum symptom severity’ (8).

Low consciousness was derived from the questions on pain, fatigue, and mood, to which a response option was added ‘do not know, the deceased had low consciousness’. These responses were considered missing for the examination of symptom severity. A dichotomous variable was derived indicating low consciousness, if low consciousness was reported for any of the three symptoms (value 1) and no low consciousness (value 0).

### 2.3 Change over time

We distinguished two time variables: historic period and within-person time. The periods in which the two samples were examined are denoted as 2008 (value 0) and 2018 (value 1). Within each period, the variable ‘within-person time’ denotes the time from 3 months before death (value 0) to 3 days before death (value 1).

### 2.4 Statistical analysis

For a descriptive overview, differences between the two periods in characteristics and end-of-life conditions of the decedents were tested using chi-square tests and analysis of variance. Because of the oversampling of older ages and men in the initial LASA-cohorts, for this overview weights were calculated to align the distribution of age at death and sex in the two study samples with those of decedents aged 60 and over in the general population in the years 2008 and 2018 (Statistics Netherlands, 2023). All subsequent analyses were unweighted but adjusted for age at death and sex in order to account for compositional differences between samples 2008 and 2018.

We then tested our hypothesis that end-of-life conditions became more protracted for non-cancer decedents between 2008 and 2018. A longer protraction would manifest as poorer health at 3 months before death in 2018 versus 2008, since decline would have been going on for a longer time already. This assumption is based on the seminal empirical study by Gill et al. (2010), in which trajectories are determined based on monthly measurements, showing a distinction between so-called catastrophic functional decline in the last 3 months on the one hand and more or less gradual, protracted functional decline on the other hand. The gradual trajectories varied in level of disability prior to 3 months before death, but these levels were always poorer than in the catastrophic decline trajectory. Thus, we performed regression analyses with each health condition at 3 months as the outcome and period as the main independent variable. Linear regression models were used for functional limitations and symptom severity, and a logistic regression model for low consciousness.

To address our hypothesis of a more precipitous change in end-of-life conditions in cancer-than in non-cancer decedents, within-person changes between 3 months and 3 days before death in end-of-life conditions were modelled using Generalised Estimating Equations. An exchangeable correlation structure was used to account for interdependency of repeated measurements within participants (Twisk, 2013). Analyses were performed for each single health condition as the dependent variable. Functional limitations and symptom severity were examined with a linear model and low consciousness, with a logistic model. Each model consisted of within-person time, period, dichotomous cause of death, three two-way interaction terms, i.e., within-person time∗period, cause of death∗period, and cause of death∗within-person time, and the three-way interaction term cause of death∗within-person time∗period. By changing the reference category of the period variable, we estimated a model for the cause of death∗within-person time interaction for each period, with the covariates having equal effects (Figueiras et al., 1998). The two-way interaction terms were tested for significance at p < 0.10 (Aiken et al., 1991). The three-way interaction term was needed for a correct model but was not tested for significance, as period differences were determined by examining whether the regression coefficients of cause of death, within-person time, and the cause of death∗within-person time interaction in one period exceeded the corresponding 95 % confidence intervals in the other period.

Because of the presence of the interaction term within-person time∗cause of death, in each model for each period, the coefficient of the main effect of cause of death represents the effect of cause of death on health for within-person time value 0, i.e., at 3 months before death. Likewise, the coefficient for the main effect of within-person time represents the health change for non-cancer decedents (value 0). For cancer decedents, health change is obtained by adding the coefficient of the interaction term cause of death∗within-person time to the coefficient of within-person time in the linear regression models, or in the logistic regression model by multiplying the odds ratios of within-person time with the odds ratio of the interaction term cause of death∗within-person time.

Our hypothesis that end-of-life changes for cancer decedents are more precipitous than end-of-life changes for non-cancer decedents, is then tested by the interaction term cause of death∗within-person time for each period. This interaction term should not only be statistically significant, but also have a positive sign, indicating a steeper health decline in cancer than in non-cancer decedents.

Our alternative hypothesis that the health changes in cancer and non-cancer decedents have become more similar involves testing if the regression coefficient of the cause of death∗within-person time interaction for 2018 is closer to zero and lies outside the corresponding 95 % confidence interval in 2008.

In all analyses, effect sizes for continuous dependent variables were calculated as the regression coefficient of independent dichotomous variables (within-person time, cause of death, their interaction, and sex), divided by the standard deviation of the dependent variable; the regression coefficient of independent continuous variables (age at death, years of education) were multiplied by their standard deviation and divided by the standard deviation of the dependent variable. Effect sizes 0.2 to 0.5 indicate a small, 0.5 to 0.8 a medium, and above 0.80 a large effect (Cohen, 1988). For dichotomous dependent variables, effect sizes were expressed as Odds Ratios (ORs), with values 1.20–1.50 or 0.67–0.83 considered to indicate a small, 1.50–1.80 or 0.56–0.67 a medium, and above 1.80 or below 0.56 a large effect.

The health changes in the last 3 months of life by cause of death are also presented visually in graphs as estimated marginal mean scores (functional limitations and symptom severity) and probabilities (low consciousness).

## 3 Results

### 3.1 Descriptive findings by period

In the weighted samples, average age at death increased by 2.5 years from 2008 to 2018 (Table 1). In both samples, almost half of the decedents were men and about half of the decedents had a partner. In 2018, level of education was over 1 year higher than in 2008, which rise was significant. Therefore, level of education was included as an additional covariate in all analytic models.

Place of death differed significantly between 2008 and 2018. The largest difference was the decline in the proportion of deaths in a hospital from 28 % to 13 %. Somewhat more participants died in a hospice or in their own home in 2018 than in 2008. Overall shifts in the proportions of causes of death were not statistically significant. However, some shifts of more than five percentage points are noteworthy. The proportion of cancer increased by six percentage points to 36 %. Stroke decreased by seven percentage points to 4 %.

Considering the health conditions, no significant differences were observed between the periods. Regardless, it may be noted that the prevalence of low consciousness at 3 months before death at 12 % was six percentage points higher in 2018 than in 2008. Correlations of functional limitations with symptom severity and low consciousness were higher in 2018 than in 2008, but not significantly so (Supplement Table S1). Averaged across samples, these correlations amounted to 0.34 and 0.23 at 3 months, respectively. At 3 days, they were higher at 0.54 and 0.32, respectively.

### 3.2 Health conditions at 3 months before death in non-cancer decedents

No differences were observed between the periods in functional limitations and symptom severity in non-cancer decedents at 3 months before death (Table 2). However, a more than two times greater odds of low consciousness was apparent in 2018 than in 2008, showing a large effect size, although the statistical significance was marginal (p = 0.062). Thus, we found some support for our hypothesis of greater protraction of health decline for low consciousness.

**Table 2:** The association of health conditions at three months before death with decade in non-cancer decedents (n ≤ 206). Linear regression (functional limitations and symptoms) and logistic regression (low consciousness) of health condition on decade, with covariates sex, age-at-death, and years of education.

Health condition	Decade		Sex (Female vs Male)		Age-at-death		Years of education	
	B (95 % CI)	Effect size^a^	B (95 % CI)	Effect size^a^	B (95 % CI)	Effect size^a^	B (95 % CI)	Effect size^a^
Functional limitations	0.04 (−1.61; 1.69)	0.004	0.34 (−1.30; 1.98)	0.03	0.20∗∗ (0.10; 0.30)	0.28	−0.17 (−0.42; 0.09)	−0.09
Symptoms	0.22 (−0.36; 0.80)	0.06	−0.28 (−0.86; 0.30)	−0.08	0.02 (−0.01; 0.06)	0.11	−0.07 (−0.16; 0.03)	−0.11

	**OR (95 % CI)**		**OR (95 % CI)**		**OR (95 % CI)**		**OR (95 % CI)**	
Low consciousness	2.38† (0.95; 5.94)		1.71 (0.67; 4.37)		0.99 (0.93; 1.04)		1.07 (0.95; 1.24)	

∗∗ p < 0.001; †p = 0.062.

a Standardised regression coefficient.

From Table 2, it can also be derived that functional limitations at 3 months were greater with increasing age at death, although the effect size is small. There were no associations with age at death for symptom severity and low consciousness.

### 3.3 Changes in health conditions

Below, we report the findings from our longitudinal models focusing on each health condition. Here, we first note that there were no associations of the covariates sex and age at death with the health conditions, other than the already noted association of age at death with functional limitations (Table 3).

**Table 3:** The association of change in health conditions during three months before death with cause of death by decade. General Estimating Equations with main effects, 2-way and 3-way interactions of decade, cause of death, and within-person time, adjusted for sex, age at death, and years of education. (The interaction terms including decade are not shown, but the significance between decades is derived by comparing the regression coefficients across samples. See also Fig. 1, Fig. 2, Fig. 3.)

	2008			2018		
A. Functional limitations (n = 284 participants, 529 observations)						
	B	95 % CI	Effect size	B	95 % CI	Effect size
**Main part of model**						
Time (3 months–3 days)	3.67∗∗	2.70; 4.64	0.58	3.23∗∗	2.29; 4.18	0.51
Cause of death (Cancer vs other)	−0.92^b^	−3.26; 1.43	−0.15	−4.48∗∗^a^	−6.71; −2.26	−0.71
Cause of death ∗ Time	4.67∗∗	2.64; 6.70	0.74	6.75∗∗^a^	4.53; 8.98	1.07
**Covariates (coefficients identical for the two decades)**						
Sex (female vs male)	0.27	−0.91; 1.45	0.04			
Age-at-death	0.14∗∗	0.06; 0.21	0.02			
Education in years	−0.16†	−0.35; 0.02	0.03			

*B. Symptom severity (n=248 participants, 389 observations)*						
	B	95 % CI	Effect size	B	95 % CI	Effect size
**Main part of model**						
Time (3 months–3 days)	1.30∗∗	0.89; 1.70	0.64	1.01∗∗	0.55; 1.46	0.50
Cause of death (Cancer vs other)	0.56	−0.13; 1.24	0.28	0.03	−0.71; 0.77	0.01
Cause of death ∗ Time	0.52	−0.30; 1.35	0.26	0.35	−0.45; 1.15	0.17
**Covariates (coefficients identical for the two decades)**						
Sex (female vs male)	−0.31	−0.75; 0.13	0.15			
Age-at-death	0.00	−0.02; 0.03	0.01			
Education in years	−0.06	−0.14; 0.02	0.10			

*C. Low consciousness (n=277 participants; 507 observations)*				
	OR	95 % CI	OR	95 % CI
**Main part of model**				
Time (3 months–3 days)	6.74∗∗	3.47; 13.10	4.00∗∗	2.23; 7.17
Cause of death (Cancer vs other)	0.25	0.03; 2.50	0.25†	0.06; 1.16
Cause of death ∗ Time	4.16	0.45; 38.47	2.17	0.46; 10.24
**Covariates (coefficients identical for the two decades)**				
Sex (female vs male)	0.92	0.55; 1.54		
Age-at-death	1.00	0.97; 1.03		
Education in years	0.98	0.90; 1.07		

B: unstandardized regression coefficient; OR: Odds Ratio; CI: Confidence Interval.

∗∗p < 0.001; ∗p < 0.05; †p < 0.10.

a Estimate differs significantly from estimate in 2008.

b Estimate differs significantly from estimate in 2018.

#### 3.3.1 Functional limitations

**Fig. 1:**
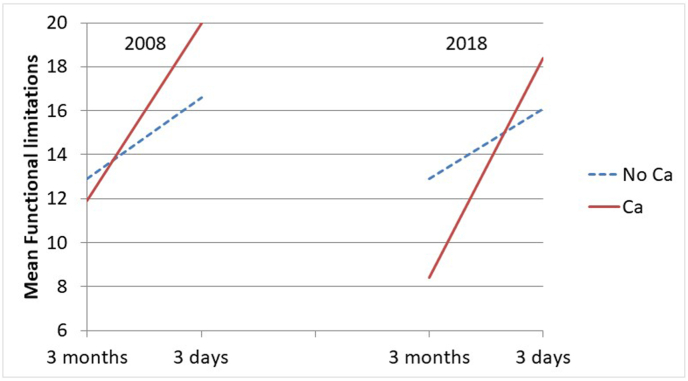
Change in functional limitations from 3 month to 3 days before death for cancer (Ca) and non-cancer (No Ca) decedents in 2008 and 2018. Marginal estimates from General Estimating Equations, adjusted for age at death, sex, and education. *Note:* Functional limitations, range 0-20*.*

Both in 2008 and 2018 the within-person time coefficients show an increase in functional limitations in non-cancer decedents between 3 months and 3 days before death, with medium effect sizes (Table 3A, first row, and Fig. 1). The rate of increase in this group was not significantly different in 2008 and in 2018, as the confidence intervals overlap. In 2008 there was no statistically significant difference in functional limitations at 3 months before death between cancer and non-cancer decedents, but in 2018, cancer decedents experienced significantly fewer functional limitations at 3 months before death than non-cancer decedents: the regression coefficient of cause of death in 2018 is higher than the upper limit of the 95 % confidence interval of the regression coefficient in 2008, and vice versa (Table 3A, second row). The effect in 2018 is of medium size. The interaction term cause of death∗within-person time is highly significantly positive in each period, with the coefficient significantly more positive in 2018 than in 2008 and with medium and large effect sizes in 2008 and 2018, respectively (Table 3A, third row). Thus, cancer decedents experienced a steeper functional decline than non-cancer decedents, and this decline started from a lower level of functional limitations in 2018 than in 2008. These findings support our hypothesis of a more precipitous decline in functioning in cancer than in non-cancer decedents. Because the decline is even steeper in 2018 than in 2008, our hypothesis of increasing similarity between cancer- and non-cancer decedents is not supported.

#### 3.3.2 Symptom severity

**Fig. 2:**
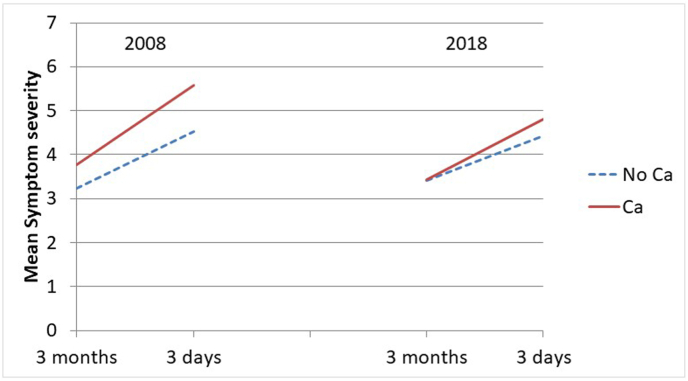
Change in symptom severity from 3 month to 3 days before death for cancer (Ca) and non-cancer (No Ca) decedents in 2008 and 2018. Marginal estimates from General Estimating Equations, adjusted for age at death, sex, and education. *Note:* Symptom severity, range 0–8.

Symptom severity in non-cancer decedents increased from 3 months to 3 days before death to a similar degree in 2008 and 2018, showing medium effect sizes (Table 3B, first row, and Fig. 2). Differences in symptom severity at 3 months between cancer- and non-cancer decedents were not significant in either period (Table 3B, second row). Furthermore, in neither period did cancer decedents experience a significantly greater increase in severity than non-cancer decedents (Table 3B, third row). These findings do not lend support to our hypothesis of a more precipitous increase in symptom severity in cancer than in non-cancer decedents. In contrast, cancer- and non-cancer decedents had quite similar trajectories in both periods and thus our hypothesis of increasing similarity is not supported either.

#### 3.3.3 Low consciousness

**Fig. 3:**
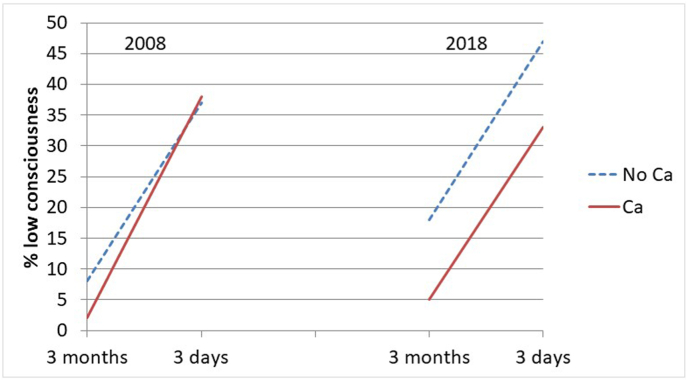
Change in prevalence of low consciousness from 3 month to 3 days before death for cancer (Ca) and non-cancer (No Ca) decedents in 2008 and 2018. Marginal estimates from General Estimating Equations, adjusted for age at death, sex, and education.

The prevalence of low consciousness in non-cancer decedents rose significantly from 3 months to 3 days before death, whereby in 2018 this rise started from a higher percentage (Table 3C, first row, and Fig. 3). In both periods, the effect sizes are large. At 3 months before death, the prevalence of low consciousness was lower in cancer decedents than in non-cancer decedents, which difference was marginally significant only in 2018 (p = 0.076), although in both periods effect sizes were large (Table 3C, second row). There was no difference in rate of increase between cancer- and non-cancer decedents in either period (Table 3C, third row). These findings do not support our hypothesis of a more precipitous health decline in cancer than in non-cancer decedents. Furthermore, because there remained a difference in prevalence of low consciousness in 2018, our hypothesis of increasing similarity is not supported either.

## 4 Discussion

The general rise in life expectancy implies that deaths occur at increasingly older ages. However, improvements in health expectancy lag behind increases in life expectancy, and thus, more years of life are spent in poor health (Spiers et al., 2021). Moreover, the prevalence of health impairments increases with age (Jenkins et al., 2022), which may result in a longer period of worsening health in the last phase of life. On the other hand, the proportion of cancer among causes of death is increasing (GBD, 2018). Because evidence shows that health decline is compressed into a shorter time period in persons dying of cancer compared to persons dying of other causes (e.g., Teno et al., 2001), it may be expected that the period of worsening health is becoming shorter. This study aimed to disentangle both developments by examining changes in various health conditions at the end of life in the late 2000s and the late 2010s, distinguishing cancer from non-cancer decedents. To do so, we formulated three hypotheses.

Our first hypothesis stated that non-cancer decedents would experience a more protracted period of poor health in the late 2010s than in the late 2000s. We found marginal evidence for such protraction only for low consciousness and not for functional limitations and symptom severity. However, the measurement of symptom severity was contingent on the level of consciousness; the proxies were not able to assess symptoms such as pain, fatigue, and mood when their relative had low consciousness. Focusing on symptoms alone provides only insight into the health condition of decedents who remain conscious. A longer period of low consciousness, therefore, can be considered to be a strong indicator of a more protracted period of poor health at the end of life.

Our second and third hypotheses addressed the differences in end-of-life changes between cancer and non-cancer decedents. The findings from earlier studies were replicated in that a more precipitous functional decline was observed in cancer than in non-cancer decedents. Interestingly, in 2018 cancer decedents had fewer functional limitations at 3 months before death as well as a steeper increase in functional limitations up to 3 days before death than in 2008. This finding suggest that the period of functional decline associated with terminal cancer has shortened. Improvements in treatment and expansion of palliative care may have contributed to this shortening (Bergman et al., 2024; Cheraghlou et al., 2016; Geijteman et al., 2024).

We did not, however, observe a difference between cancer- and non-cancer decedents in rate of increase in symptom severity and low consciousness. This would tie in with our alternative hypothesis stating an increase in similarity of end-of-life changes for cancer and non-cancer decedents over time. Yet, we did not find an increase in similarity across the two periods. Instead, similarity was apparent in both periods to the same extent. The fact that we did not observe the expected change from 2008 to 2018, may be explained by the relatively small increase in age at death in our study. Future studies may be able to address end-of-life changes over longer periods of time, with greater shifts in the age at death.

The studies that address trajectories in cancer and non-cancer decedents focus on disability (Teno et al., 2001; Lunney et al., 2003; Gill et al., 2010; Stolz et al., 2021) or health care utilisation (Ebeling et al., 2023; Stolz et al., 2024). Despite differences in design and outcome measures, these studies provide ample support for a more precipitous decline in cancer than in non-cancer decedents. Regardless, studies in cancer decedents alone show variation in trajectories. For example, Soeda et al. (2024) distinguished four ADL disability trajectories across eight weeks, from stable good ADL (21 %), rapidly declining ADL (19 %), moderately declining ADL (34 %) to stable severely limited ADL (26 %). How the variation in trajectories changes over time is to be examined in future research.

Earlier research on time trends of end-of-life conditions is limited and focuses on the prevalence of conditions in periods of varying length before death. Using the U.S. Health and Retirement Study (HRS), Beltrán-Sánchez et al. (2016) compared the periods 1998–2004 and 2004–2010 in age-matched decedents. Over time, they found a higher prevalence in seven of eight chronic conditions, including cancer, but not in lung disease. Vierboom (2022) selected participants in the U.S. National Health Interview Survey who had died within six years of being interviewed, with their last interview during the years 1997–2008. While there was no change in disability prevalence in men, in women disability prevalence increased by 2008. In a Danish study using prescription medication registries in the last year of life, Pedersen et al. (2020) observed an increase from 1995 to 2012 in medication use in age-matched cohorts aged 90 and over, with substantially larger use in the most recent cohort. Singer et al. (2015), using after-death proxy reports of symptoms in the HRS, reported an increase in four out of eight symptoms one year before death from 1998 to 2010 for causes of death other than cancer. In cancer decedents, no change was observed over time. In contrast, Gill and colleagues (2021) reported an overall decrease in 16 self- or proxy-reported symptoms during the last 18 months of life between 1998 and 2019. However, separate symptoms showed different changes over time. Amongst others, shortness of breath decreased, whereas fatigue and mood showed no change.

These studies evidence an increase in morbidity and disability at the end of life in more recent years, but not necessarily in symptoms. This corresponds to our finding of greater functional limitations at 3 months prior to death with increasing age at death. We also found a stronger correlation between functional limitations and symptoms at 3 days than at 3 months before death, implying that health conditions are increasingly intertwined. Studies focusing on one health condition only may miss important changes at the end of life.

### 4.1 Strengths and limitations

Our study has several strengths. First, we examined within-person change as retrospectively reported by proxies, whereas many studies combined data from different decedents to construct trajectories over the last years or months of life (Kristensen et al., 2022; Lunney et al., 2003; Smith et al., 2013; Teno et al., 2001). Second, although the period from three months to three days before death is shorter than most studies, it captures a critical period particularly in cancer decedents. A period of three months before death has been reported to be the duration of the final stage in the end-of-life period, with the greatest health decline regardless of cause of death (Cohen-Mansfield et al., 2018) and has been shown to reflect most of the decline in cancer decedents (Kristensen et al., 2022; Lunney et al., 2003; Versluis et al., 2024). Yet, the availability of only two time points does not allow investigating nonlinear, accelerated change in health conditions.

Another strength is that we examined both functioning and symptoms, whereas most studies either examine functional limitations or disability only (Edjolo et al., 2020; Gill et al., 2010; Lunney et al., 2003; Stolz et al., 2021; Teno et al., 2001) or symptoms only (Cheraghlou et al., 2016; Gill et al., 2021; Singer et al., 2015). Some other studies are registry-based so that health care use is considered a proxy for health (Ebeling et al., 2023; Pedersen et al., 2020; Stolz et al., 2024). Our choice of measures aligns with an extensive review of outcome measures in end-of-life care (Kawashima & Evans, 2023) recommending needs-based outcome measures, such as functional status and symptoms, rather than diagnostic criteria or medical history.

A further strength is the inclusion of consciousness. Studies on end-of-life symptoms generally use self- or proxy reports, which presume consciousness of the participant. As we have shown in this study, low consciousness increased with approaching death as well as across periods, so that a comparison of symptoms over time are hampered by increased selectivity. Thus, comparisons of end-of-life symptoms over time are likely to be positively biased. This, of course, is also a limitation of our own study.

Our findings should also be considered in the light of several further caveats. First, after-death proxy-reports of functional limitations and symptoms may suffer from recall bias. Recall bias may involve under- or overestimation of health conditions, but also of exact timing of when a condition was present. Additionally, proxy reporting behaviour may change over time, for example because higher levels of educational attainment in more recent cohorts may lead to increased knowledge and understanding of one's relative's health, influencing awareness and reporting propensity (Parker & Thorslund, 2007). Although our analyses were adjusted for educational level, increased awareness may residually affect the level of reported health, but it is unlikely to affect the report of change from 3 months to 3 days before death. In a study comparing proxy reports of symptoms with self-reports by the decedents at earlier waves in the study and with reports by general practitioners, the symptoms pain and shortness of breath showed fair concordance between proxy and general practitioner reports, but mood showed low concordance (Klinkenberg et al., 2003). A more recent study by Kroenke et al. (2022) also showed relative similarity between caregiver and patient reports, although concordance was better for pain than for mood. In any case, proxy reports are likely to contribute essential information in decision making about end-of-life care (Gerlach et al., 2017; Kawashima & Evans, 2023).

A second caveat is the relatively small size of our samples, for example prohibiting the separate study of male and female decedents. Amongst others Vierboom (2022) observed a sex difference in that women had greater disability at the end of life. Also, we studied average change within cause-of-death subsamples and it was not possible to distinguish different trajectories, so that heterogeneity in trajectories is missed. For example, Gill et al. (2010) showed substantial heterogeneity in cancer-decedents. We recommend that future studies with larger samples available address changes over time in end-of-life trajectories.

A third caveat concerns potential differences in participation histories between samples 2008 and 2018, which may bias any comparison. Participation histories concern initial participation rates and non-mortality attrition rates. The three successively recruited LASA cohorts maintained consistent initial response rates (62–63 %) and low non-mortality attrition (5.5 % on average). Non-mortality attrition decreased across waves, but was increasingly higher in the second and particularly the third cohort, compared to the first cohort (Hoogendijk et al., 2025). However, both samples predominantly belonged to the first cohort (90 % in 2008, 74 % in 2018), with only 5 % of the 2018 sample belonging to the third cohort. The difference in participation rate histories in the two samples, therefore, is likely to be small and will minimally affect our comparisons.

Finally, we adjusted all analyses for age at death in order to adjust for compositional changes between the 2008 and 2018 samples. However, the rise in average age at death was also one of the reasons why we expected differences in end-of-life trajectories, and including it in our models might have obscured important period differences. Therefore, we reran our analyses omitting age at death (Supplement Table S2–S3, Supplement Fig. S1). The results were virtually the same as from the original analyses.

### 4.2 Conclusion

Our study is, to our best knowledge, the first to compare health changes within three months before death in older people across ten years. Thereby, it distinguishes cancer and non-cancer decedents and includes a variety of health indicators. As such, it contributes to the literature on trajectories at the end of life. We found that in non-cancer decedents, the dying process was more protracted in 2017–2019 than in 2005–2009. At the population level, this finding goes against suggestions of compression of morbidity (Fries, 1980), as it implies that life expectancy in poor health has increased. By contrast, cancer decedents experienced a more precipitous functional decline than non-cancer decedents, even more so in 2017–2019 than in 2005–2009. As an increasing proportion of people dies from cancer, the end-of-life trajectory of functional decline will be shorter in the average population of decedents. At the population level, this would imply that the expected duration of life with disability has decreased. Meanwhile, in cancer decedents the end-of-life trajectory of symptom severity tends to be similar to that of non-cancer decedents, with no substantial change across the decades. Future research may more closely focus on the connection between trends in end-of-life trajectories in decedents and trends in healthy life expectancy in the general population.

While it is often difficult to predict the timing of death, the ability to recognize the clinical signs and symptoms of imminent death in terminally-ill individuals may lead to earlier anticipation of care needs and better planning to provide care that is tailored to individuals’ needs (Ijaopo et al., 2023; Murray, 2024). Our study adds important insights into the way such signs and symptoms change over time, necessitating the continuous monitoring of the adequacy of existing care provision plans.

## Authorship contribution statement

**Dorly J.H. Deeg:** Writing – review & editing, Writing – original draft, Visualization, Methodology, Formal analysis, Data curation, Conceptualization. **H. Roeline W. Pasman:** Writing – review & editing, Data curation, Conceptualization. **Martijn Huisman:** Writing – review & editing, Conceptualization. **Bregje D. Onwuteaka-Philipsen:** Writing – review & editing, Data curation, Conceptualization.

## Ethical statement

The Amsterdam University Medical Centre's Medical Ethics Evaluation Committee approved the study (archive numbers 92/138 and 2002/141). Signed informed consent was obtained from all study participants. All methods were carried out in accordance with the Helsinki declaration.

## Funding

The Longitudinal Aging Study Amsterdam is supported by grants from the Netherlands Ministry of Health Welfare and Sports, Directorate of Long-Term Care.

## Declaration of competing interest

None declared.

## Data Availability

The dataset generated and analysed is available for replication purposes, provided an agreement is made up. Please see www.lasa-vu.nl.
