# Supplementary material for: Two decades of end-of-life conditions of older adults: older and more protracted?
Source: SSM Popul Health. 2025 Aug 30;31:101858. doi: 10.1016/j.ssmph.2025.101858 (PMC12424242; doi:10.1016/j.ssmph.2025.101858)
Supplement: Multimedia component 1 [file mmc1.docx]

**Appendix A. Supplement to**

**“Two decades of end-of-life conditions of older adults: older and more protracted?”**

Dorly J.H. Deeg, H. Roeline W. Pasman, Martijn Huisman, Bregje D. Onwuteaka-Philipsen

**Table S1. Correlations with confidence intervals of functional limitations with symptom severity and low consciousness^a^ at 3 months and 3 days before death in two decades**

|  | **2008** | **2018^c^** |
| --- | --- | --- |
| Functional limitations – symptom severity at 3 months | 0.30 (0.14-0.44) | 0.42 (0.25-0.57) |
| Functional limitations – symptom severity at 3 days^b^ | 0.52* (0.34-0.66) | 0.57† (0.35-0.73) |
| Functional limitations – low consciousness at 3 months | 0.18 (0.03-0.33) | 0.31 (0.13-0.46) |
| Functional limitations – low consciousness at 3 days | 0.26 (0.10-0.42) | 0.40 (0.21-0.56) |

^a^ Correlations between symptom severity and low consciousness are not possible by definition

^b^ Correlation significantly greater at 3 days than at 3 months, * p<0.05, † p<0.10

^c^ All correlations are greater in 2018 than in 2008, but never significantly so

**Table S2**. The association of health conditions at three months before death with decade in non-cancer decedents (n<=206). Linear regression (functional limitations and symptoms) and logistic regression (low consciousness) of health condition on decade, with covariates sex and years of education.

|  | **Decade** | | **Sex (Female vs Male)** | | **Years of education** | |
| --- | --- | --- | --- | --- | --- | --- |
| **Health condition** | **B (95% CI)** | **Effect size****^a^** | **B (95% CI)** | **Effect size^a^** | **B (95% CI)** | **Effect size^a^** |
| Functional limitations | 0.54 (-1.16; 2.23) | 0.04 | 0.56 (-1.14; 2.26) | 0.03 | -0.23 (-0.50; 0.03) | -0.13 |
| Symptoms | 0.27 (-0.31; 0.84) | 0.07 | -0.24 (-0.81; 0.34) | -0.06 | -0.07 (-0.17; 0.02) | -0.12 |
|  | **OR (95% CI)** | | **OR (95% CI)** | | **OR (95% CI)** | |
| Low consciousness | 2.29† (0.93; 5.63) | | 1.67 (0.66; 4.23) | | 1.09 (0.96; 1.24) | |

** p<0.001; † p=0.07

^a^ Standardised regression coefficient **Table S3**. The association of change in health conditions during three months before death with cause of death by decade. General Estimating Equations with main effects, 2-way and 3-way interactions of decade, cause of death, and within-person time, adjusted for sex and years of education. (The interaction terms including decade are not shown, but the significance of differences between decades is derived by comparing the regression coefficients across samples. See also Figures 1-3.)

|  | **2008** | | | **2018** | | |
| --- | --- | --- | --- | --- | --- | --- |
| ***A. Functional limitations (n=284 participants, 529 observations)*** | | | | | | |
|  | **B** | **95% CI** | **Effect size** | **B** | **95% CI** | **Effect size** |
| **Main part of model** | | | | | | |
| Time (3 months to 3 days) | 3.68** | 2.71; 4.66 | 0.58 | 3.24** | 2.31; 4.18 | 0.51 |
| Cause of death (Cancer vs other) | -1.93†^b^ | -4.02; 0.17 | -0.31 | -5.23**^a^ | -7.42; -3.05 | -0.83 |
| Cause of death * Time | 4.62** | 2.60; 6.66 | 0.73 | 6.79**^a^ | 4.58; 8.99 | 1.07 |
| **Covariates (coefficients identical for the two decades)** | | | | | | |
| Sex (female vs male) | 0.24 | -0.98; 1.46 | 0.04 |  | | |
| Education in years | -0.20* | -0.40; -0.01 | 0.03 |  | | |
| ***B. Symptom severity (n=248 participants, 389 observations)*** | | | | | | |
|  | **B** | **95% CI** | **Effect size** | **B** | **95% CI** | **Effect size** |
| **Main part of model** | | | | | | |
| Time (3 months to 3 days) | 1.29** | 0.89; 1.70 | 0.64 | 1.01** | 0.55; 1.46 | 0.50 |
| Cause of death (Cancer vs other) | 0.53 | -0.10; 1.16 | 0.26 | 0.01 | -0.71; 0.74 | 0.01 |
| Cause of death * Time | 0.52 | -0.31; 1.35 | 0.26 | 0.35 | -0.44; 1.15 | 0.17 |
| **Covariates (coefficients identical for the two decades)** | | | | | | |
| Sex (female vs male) | -0.31 | -0.75; 0.13 | 0.15 |  | | |
| Education in years | -0.06 | -0.14; 0.02 | 0.10 |  | | |
| ***C. Low consciousness (n=277 participants; 507 observations)*** | | | | | | |
|  | **OR** | **95% CI** | | **OR** | **95% CI** | |
| **Main part of model** | | | | | | |
| Time (3 months to 3 days) | 6.74** | 3.47; 13.10 | | 3.99** | 2.22; 7.17 | |
| Cause of death (Cancer vs other) | 0.26 | 0.03; 2.42 | | 0.25† | 0.06; 1.16 | |
| Cause of death * Time | 4.16 | 0.45; 38.45 | | 2.17 | 0.46; 10.23 | |
| **Covariates (coefficients identical for the two decades)** | | | | | | |
| Sex (female vs male) | 0.92 | 0.55; 1.54 | |  | | |
| Education in years | 0.98 | 0.90; 1.07 | |  | | |

B: unstandardized regression coefficient; OR: Odds Ratio; CI: Confidence Interval

** p<0.001; * p<0.05; † p<0.10

^a^ Estimate differs significantly from estimate in 2008

^b^ Estimate differs significantly from estimate in 2018
